# Biomarker Changes Associated with Tuberculin Skin Test (TST) Conversion: A Two-Year Longitudinal Follow-Up Study in Exposed Household Contacts

**DOI:** 10.1371/journal.pone.0007444

**Published:** 2009-10-14

**Authors:** Rabia Hussain, Najeeha Talat, Firdaus Shahid, Ghaffar Dawood

**Affiliations:** 1 Department of Pathology and Microbiology, The Aga Khan University, Karachi, Pakistan; 2 Masoomeen General Hospital, Kharadar, Karachi, Pakistan; MRC Laboratories, Gambia

## Abstract

**Background:**

A high prevalence (50–80%) of Tuberculin Skin Test Positivity (TST+ ≥10 mm indurations) has been reported in TB endemic countries. This pool forms a huge reservoir for new incident TB cases. However, immune biomarkers associated with TST conversion are largely unknown. The objective of this study was to identify immune biomarkers associated with TST conversion after acute *Mycobacterium tuberculosis* (MTB) exposure.

**Methodology/Principal Findings:**

A 24 month longitudinal study was carried out in a recently MTB exposed cohort of household contacts (HC = 93; 75% TST+). Control group consisted of unexposed community controls (EC = 59; 46%TST+). Cytokine secretion was assessed in whole blood cultures in response to either mycobacterial culture filtrate (CF) antigens or mitogens (PHA or LPS) using Elisa methodology. Compared to the EC group, the HC group at recruitment (Kruskal-Wallis Test) showed significantly suppressed IFN γ (p = 0.0001), raised IL-10 (p = 0.0005) and raised TNF α (p = 0.001) in response to CF irrespective of their TST status. Seventeen TST-HC, showed TST conversion when retested at 6 months. Post TST conversion (paired t tests) significant increases were observed for CF induced IFN γ (p = 0.038), IL-10 (p = 0.001) and IL-6 (p = 0.006). Cytokine responses were also compared in the exposed HC group with either recent infection [(TST converters (N = 17)] or previous infection [TST+ HC (N = 54)] at 0, 6, 12 and 24 months using ANOVA on repeated measures. Significant differences between the exposed HC groups were noted only at 6 months. CF induced IFN γ was higher in previously infected HC group (p = 0.038) while IL-10 was higher in recently infected HC group (p = 0.041). Mitogen induced cytokine secretion showed similar differences for different group.

**Conclusions/Significance:**

Our results suggest that TST conversion is associated with early increases in IFN γ and IL-10 responses and precedes latency by several months post exposure.

## Introduction

Pakistan ranks 8^th^ in terms of global tuberculosis (TB) disease burden [1]. One third of the world population is considered to be latently infected as assessed by Tuberculin Skin Test positivity (TST+ ≥10 mm of indurations) [2] resulting in a huge reservoir for new incident cases. In a high transmission setting such as Pakistan the rate of TST positivity can be as high as 50% in the community[3] and household contacts [4] increasing up to 80% in recently exposed household contacts [5], [6]. PPD administered in TST is a mixture of proteins prepared from *M. tuberculosis (MTB)* culture filtrate and is highly cross reactive with other mycobacterial antigens including *Mycobacterium bovis*. Pakistan has wide BCG vaccination coverage at birth (>70%) through the expanded program for immunization (EPI) [7] and the high prevalence of TST+ in the community may be due to either previous BCG vaccination [8] or booster effects with environmental mycobacteria [9]. ESAT-6, induced interferon (IFN γ) is considered to be a MTB specific marker for infection and disease as it is present only in the *M. tuberculosis* complex [10]. Commercial IFNγ release assays (IGRA) using ESAT-6 stimulated whole blood cultures are increasingly available for detection of recent infection [11] or disease [12]. However, longitudinal studies suggest IGRA test cannot replace TST in high TB incidence areas for detection of latent infection [13]. The high incidence of HIV (∼11%) in 8.8 million new TB cases [14] further confounds the immune correlates associated with latency as immune suppression results in lower TST diameters in HIV+ individuals with latent TB infection. There is an urgent need to carry out such studies in TB endemic areas with a low HIV prevalence to understand unmitigated immune correlates associated with TST conversion. Immune activation after a recent exposure may be associated with activation and modulation of several immune markers and the magnitude and timing of pro- and down-regulatory cytokines may be very important for TST conversion during a natural exposure. IFN γ is a known correlate of delayed type hypersensitivity as well as TST skin test diameter [15]. Early activation of IFN γ is therefore linked to TST conversion and development of effective immunity. TNFα in conjunction with IFN γ has been shown to play a role in granuloma formation [16] and in maintenance of granuloma [17]. IL-10 is a down-regulatory cytokine and plays a key role in limiting pathology by inhibiting overproduction of pro-inflammatory cytokines [18], [19] particularly in the chronic phase of infection [20], [21]. IL-6 is a pro-inflammatory cytokine secreted by both macrophages and T cells and plays a critical role in driving the differentiation of B cells [22]. In the mouse gene knockout model IL-6 has also been shown to participate in the early induction of IFN γ production [23]. We have therefore opted to analyze these biomarkers post acute exposure due to their identified roles in control of mycobacterial infections. Due to slow replication of mycobacteria, immune responses are usually slow to evolve requiring serial testing over several months post infection. Longitudinal studies for immune correlates of tuberculosis infection and disease have been carried out in African countries with relatively high HIV prevalence [24]–[27]. Pakistan still has a relatively low HIV (0.8/100000) prevalence [1] compared to 1–3% in eastern Africa and 10.8% in South Africa [28] and therefore provides a useful setting for such studies. The objective of the current study was to evaluate multiple interrelated biomarkers serially in a MTB exposed cohort to identify biomarkers associated with TST conversion and to analyze differences in the evolution of biomarkers in household contacts with either recent or past infection post acute exposure.

## Materials and Methods

The study cohort comprised of healthy household contacts (HC = 93) from 20 families. Each family had at least one recently diagnosed, untreated pulmonary tuberculosis patient. The intensity and duration of exposure as assessed by history of symptoms and the extent of disease in the index case was similar in all families (Table S1). Inclusion criteria: household contacts who had no previous history of anti-tuberculous treatment (ATT) and who remained disease-free during a 24 month follow up period [5], [29]. Exclusion criteria: Children under the age of five or subjects with underlying diseases which could result in immune suppression or individuals on steroids. Endemic controls (EC, N = 59) were employees working in various capacities at The Aga Khan University with no history of recent exposure to tuberculosis. Lady Health Visitors (LHV) made home visits, obtained informed written consent from all adult participants or guardians in case of children <18 years. There is no routine testing for HIV in Pakistan because of the low incidence [1].

### Tuberculin Skin Tests

Tuberculin Skin Tests (TST) were carried out by injecting 5 tuberculin units intra-cutaneously in the volar surface of the arm. A single tester administered and read the indurations after 48 hours using a caliper. A diameter≥10 mm was considered positive [30]. At intake TST was carried out on all HC (N = 93) and EC (N = 59). Repeat testing was carried out in TST-HC (N = 23). No further TST testing was done on the TST+HC group and no prophylactic treatment was provided for latent tuberculosis infection.

### Reagents

Purified protein derivative from *Mycobacterium tuberculosis* (MTB) was obtained from Staten Serum Institute (batch RT47; Copenhagen, Denmark). LPS and PHA were purchased from Sigma (St. Louis MO, USA). MTB Culture Filtrate (CF) proteins were prepared in-house at Case Western Reserve University, Cleveland, Ohio and provided by Dr Robert Wallis as described previously [31]. Briefly MTB strain H37 Rv was grown in Prouskeur Beck medium. After 8–10 weeks of culture, bacilli were removed by sedimentation and filtration. The preparation contained minimal amounts of endotoxin as determined by inhibition with polymyxin B [32]. Pairs of mouse monoclonal antibodies specific for each cytokine were purchased from Pharmingen (San Diego, CA) for the assessment of cytokines. All reagents were used according to manufacturer's instructions.

### Whole blood stimulation assay

Whole blood stimulation assay has been described in detail previously [33]. Briefly, 5 ml of blood collected by venipuncture from each donor was mixed with sodium heparin (20 U/ml; Leo pharmaceutical, Ballerup, Denmark) in 15 ml plastic centrifuge tubes (BD Falcon). Blood was further diluted 1∶10 with RPMI 1640 tissue culture medium containing 100 U/ml of penicillin+100 µg/ml of streptomycin (Sigma Chemicals St. Louis, MO. USA) and 2 mM of L-glutamine(Sigma Chemicals). Diluted blood (900 µl/well) was dispensed in 24-well tissue culture plates (Flow laboratories, Irvine, CA) within two hours of collection. The blood was subsequently stimulated with 100 µl of PHA [5.0 µg/ml] or LPS [1.0 µg/ml] or CF [5.0 µg/ml] in tissue culture plates and further incubated at 37°C in 5% CO_2_. Blood cultures were stimulated in separate wells for either 2 days (TNF α, IL-10 and IL-6) or 5 days (IFN γ). Supernatants of whole blood cultures were collected and stored as 4×200 µl of aliquots at −35°C until use. Selection of mitogen/antigen concentrations and optimal days for collection of supernatants for various cytokines has been reported previously [33].

### Cytokine assessment

The Elisa assay was optimized in-house and details of the assay have been reported previously [33]. Briefly Immulon 4 plates were coated with capture antibodies and subsequently incubated with 100 µl of un-stimulated or stimulated culture supernatants overnight at 4°C. Subsequently the probing and revealing antibodies were added (100 µl) stepwise for appropriate incubations. The plates were washed between incubations. All probing antibodies were biotin labeled and the revealing antibodies were labeled with avidin bound to horse- radish peroxidase (HRP) (Sigma St Louis MO). HRP substrate was added and color development was stopped with 1N NaOH after 30 minutes incubation. The optical densities were read using an Elisa reader (Biorad microplate reader 680). Mitogen stimulated cytokines were assessed at a dilution of 1∶10 and 1∶100 and antigen stimulated cytokines were assessed at neat and a dilution of 1∶10 of the supernatants. Optical density values falling in the midrange of the standard curve were used to calculate the concentration of cytokine in the supernatants and final values were obtained after multiplying by the dilution at which the cytokines were assessed. In case of PHA stimulated cultures occasionally (IFN γ in 3 donors) a dilution of 1∶1000 was needed to calculate the final value. Results were expressed as δ pg/ml, after deducting secretion in the absence of stimulus to normalize the expression of mitogen/antigen specific secretion. The sensitivity and range of cytokine detection was as follows: TNF α (7.8–1000 pg/ml); IL-6 (7.8–1000 pg/ml); IL-10 (15–250 pg/ml); Interferon γ (50–2000 pg/ml).

### Statistical analysis

SPSS soft ware (version 16.0) was used for the statistical analyses. Kruskal-Wallis analysis was carried to compare overlapping test groups with control group. Paired student t tests were applied to compare two related samples. Analysis of variance (ANOVA) on repeated measures was carried out using General Linear Model (GLM) for comparison of group cytokine profiles.

### Ethics statement

The study protocol received approval of the Ethical Review Committee of The Aga Khan University and Hospital.

## Results

### Study subjects

Table 1 shows the characteristics of the study groups at recruitment (0 months). The EC group was healthy and had no co-morbid conditions that could compromise the immune response. The intensity and duration of *Mycobacterium tuberculosis* (MTB) exposure was similar in the household contacts (HC) (Table S1). Although it is difficult to exclude TB exposure in high TB burden countries, none of the community controls (EC) gave a history of recent exposure to a tuberculosis patient and were considered unexposed. TST diameter in the TST+HC and the TST+EC groups was comparable (Table 1). The TST positivity in the EC group in the absence of MTB exposure is therefore most likely due to either previous BCG vaccination or exposure to environmental mycobacteria. The TST positivity in the HC group at recruitment is more likely due to previous infection as these individuals were from a high transmission pocket [6]. We have therefore, considered TST+HC group as having latent infection at the time of recruitment for the current study. The TST-HC group showed slightly higher TST diameter (mean indurations 3.5±3.0) compared to the TST-EC group (mean indurations 0.8±2.3). The absence of TST positivity in the HC group (23/93) was not related to BCG vaccination as there was no difference in the presence of BCG scar in TST-HC (40%) compared to TST+HC (38.5%) (Table S1). The slightly higher TST diameter in the TST-HC is probably due recent exposure. The TST- HC group was therefore considered a recently infected group.

**Table 1:** Characteristics of the study group at intake.

Group ID*	n	Age (X±1SD)	Male/Female	Diameter of indurations (X±1SD)
TST+HC	70	25.8±15.9	34/36	17.6±6.6
TST-HC	23	20.0±10.7	11/12	3.5±3.2
TST+EC	27	31.3±9.4**	10/17	16.6±4.1
TST-EC	32	24.0±5.0	18/14	0.8±2.3

* HC = Healthy household contacts. EC = Community controls.

TST+ = Positive Tuberculin Skin Test diameter ≥10 mm.

TST- = Negative Tuberculin Skin Test diameter <10 mm.

** One way ANOVA, p = .008.

### Cytokine secretion in recently exposed HC and non exposed EC in mycobacterial antigen (CF) stimulated whole blood cultures

**Figure 1:**
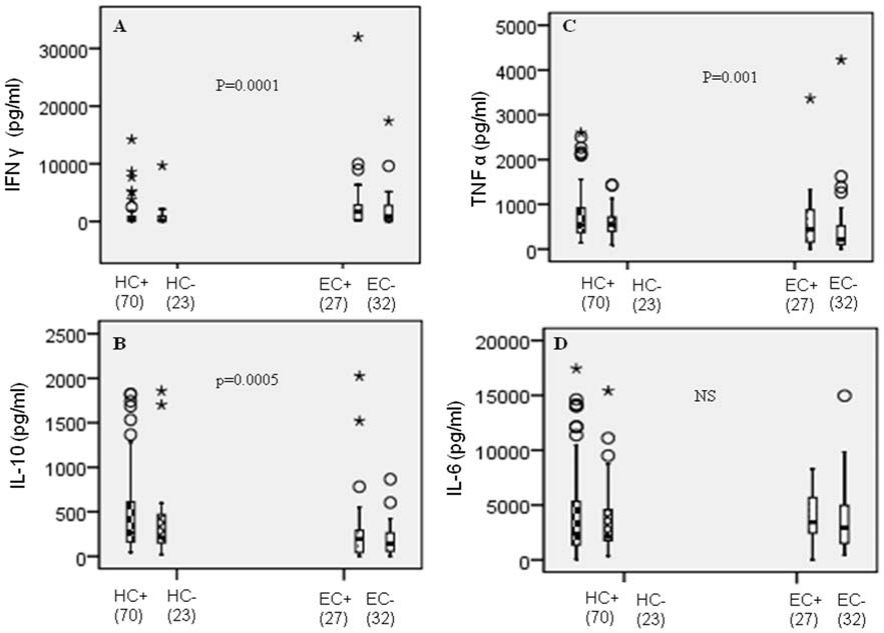
Comparison of CF induced cytokine responses in MTB exposed HC and unexposed EC. Cytokine secretion was assessed in supernatants of stimulated whole blood cultures. CF [5 µg/ml] induced IFNγ secretion was assessed at day 5 and CF induced TNF α, IL-10 and IL-6 secretion was assessed at day 2 post- stimulation. Results are expressed after deducting background secretion in the absence of stimulation. Results are given as box plots representing 25^th^, 50^th^ and 75^th^ percentiles (log_n_ pg/ml). The number of donors in each group is given in brackets. Significant differences between the HC and EC groups for different cytokines (ANOVA; Kruskal-Wallis test) are given as p values in Figure 1.

To assess early changes in biomarkers associated with recent exposure to MTB, we first compared CF induced cytokine responses in the exposed HC group (TST-HC = 23; TST+HC = 70) with the unexposed EC (TST-EC = 32; TST+EC = 27) group (Figure 1 and Table 2). Kruskal-Wallis ANOVA tests were applied to determine the significance of differences in cytokines in the exposed HC groups compared to the non exposed EC groups. The distribution of CF induced cytokines is shown as a box plot (Figure 1) with the median and inter-quartile ranges (IQR) shown in Table 2. CF induced IFN γ was significantly suppressed (p = 0.0001) in the HC group irrespective of the TST status. Increased CF induced TNF α (p = 0.001) and CF induced IL-10 (p = 0.0005) were observed in the HC group compared to the EC groups (Table 2). No difference in CF induced IL-6 secretion was observed in the two groups. Thus the hallmarks of recent infection may be suppressed T cells and unregulated macrophage response.

**Table 2:** Cytokine secretion in recently exposed HC and non exposed EC in mycobacterial antigen induced whole blood cultures.

Groups	TST- HC	TST+HC	TST-EC	TST+EC	p =
Biomarker (pg/ml)	median (IQR)	median (IQR)	median (IQR)	median (IQR)	
IFNγ	397(1009)	283 (758)	873(2724)	1720(2619)	0.0001
TNFα	**544(452)**	**541 (574)**	216(420)	441(750)	0.001
IL-10	**212(351)**	**263(472)**	141(212)	195(258)	0.0005
IL-6	2078(2913)	2232(3932)	2918(3697)	3429(3351)	>0.1

Note: Values in bold signify significant up regulation and values underlined indicate significant suppression Kruskal Wallis analysis were applied for statistical significance.

### Comparison of mycobacterial antigen (CF) and mitogen (PHA or LPS) stimulated cytokine levels pre- and post TST conversion in exposed disease free household contacts (HC)

Repeat skin testing was carried out in 17/23 TST-HC donors at 6 months. TST conversion was noted in all donors (N = 17; TST diameters pre-conversion, mean = 3.18±3.55 SD; post conversion, mean = 11±0.6 SD mm). The remaining donors (6/23) could only be retested at 24 months and were excluded from these analyses as the time of TST conversion could not be ascertained. To assess biomarker changes associated with TST conversion, we analyzed mycobacterial antigens or mitogen induced cytokine secretion pre- and post TST conversion.

**Figure 2:**
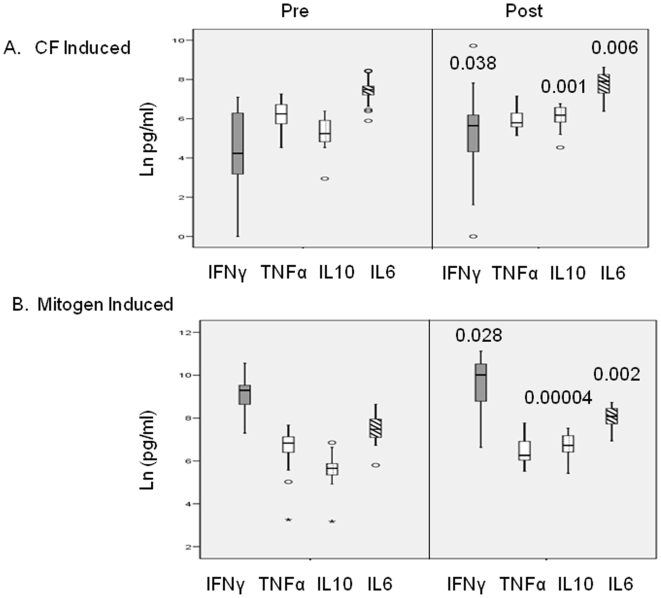
Comparison of CF and mitogens induced cytokine secretion pre- and post TST conversion. Cytokine secretion was assessed in supernatants of stimulated whole blood cultures. IFNγ secretion was assessed at day 5 and TNF α, IL-10 and IL-6 secretion at day 2 post stimulation in whole blood culture supernatants. Panel A. CF [5 µg/ml] stimulated cytokine secretion. Panel B. PHA [5 µg/ml] stimulated IFNγ and LPS [1 µg/ml] stimulated TNF α, IL-10 and IL-6 secretion. Results are given after deducting background secretion in the absence of stimulation and are depicted as box plots representing 25^th^, 50^th^ and 75^th^ percentiles (log _n_ pg/ml) on the Y axis. Paired t test were applied to determine the significance of differences between pre- and post TST conversion (N = 17) and p values for each cytokine is given.

- **Mycobacterial antigen induced cytokine secretion in HC pre- and post TST conversion.** Figure 2 (Panel A) shows a comparison of CF induced cytokine responses (log_ n_) in TST-HC group pre- and post TST conversion (N = 17). Significant increases (paired t tests) in median values were observed for CF induced IFN γ (pre- 4.30±0.51; post 5.19±0.55; p = 0.038); CF induced IL-10 (pre- 5.25±0.20; post 6.07±0.14; p = 0.001) and CF induced IL-6 (pre- 7.40±0.17; post 7.77±0.15; p = 0.006). The only cytokine that was not significantly different post TST conversion was CF induced TNF α (pre- 6.11±0.19; post 5.95±0.14; p = 0.43).
- **Mitogen induced cytokine secretion in HC pre- and post TST conversion.** We also analyzed changes in cytokine secretion in response to mitogenic stimuli pre- and post TST conversion which would reflect non-specific cellular activation. While it is difficult to define the cellular source of cytokine in whole blood cultures we have used PHA as a pan T cell activator and LPS which is potent activator of monocytes/macrophages. Mitogen stimulated cytokine pre- and post TST conversion (Figure 2, panel B) also showed significant differences with the same three cytokine [PHA induced IFN γ (pre- 9.09±0.18; post 9.63±0.28; p = 0.028); LPS induced IL-10 (pre- 5.63±0.20; post 6.70±0.13; p = 0.0004) and LPS induced IL-6 (pre- 7.50±0.17; post 8.04±.11; p = 0.002)]. LPS induced TNF α again showed comparable responses for post TST conversion (pre- 6.50±0.25; post 6.51±0.15; p = 0.96). As expected the overall levels of PHA induced IFN γ secretion was 15–18 fold higher compared to mycobacterial antigen induced IFN γ. On the other hand, LPS induced TNFα (a prototypic marker of macrophage activation) was only 2–3 fold higher compared to CF induced TNFα. These results are in line with previously published reports that mycobacterial antigens are potent stimulators of macrophages [31], [34]. The overall cellular activation was parallel to mycobacterial antigen stimulated cytokine secretion.

### Longitudinal assessment of CF and mitogen induced cytokine secretion post exposure in recently infected and previously infected HC groups over a 2-year period

**Figure 3:**
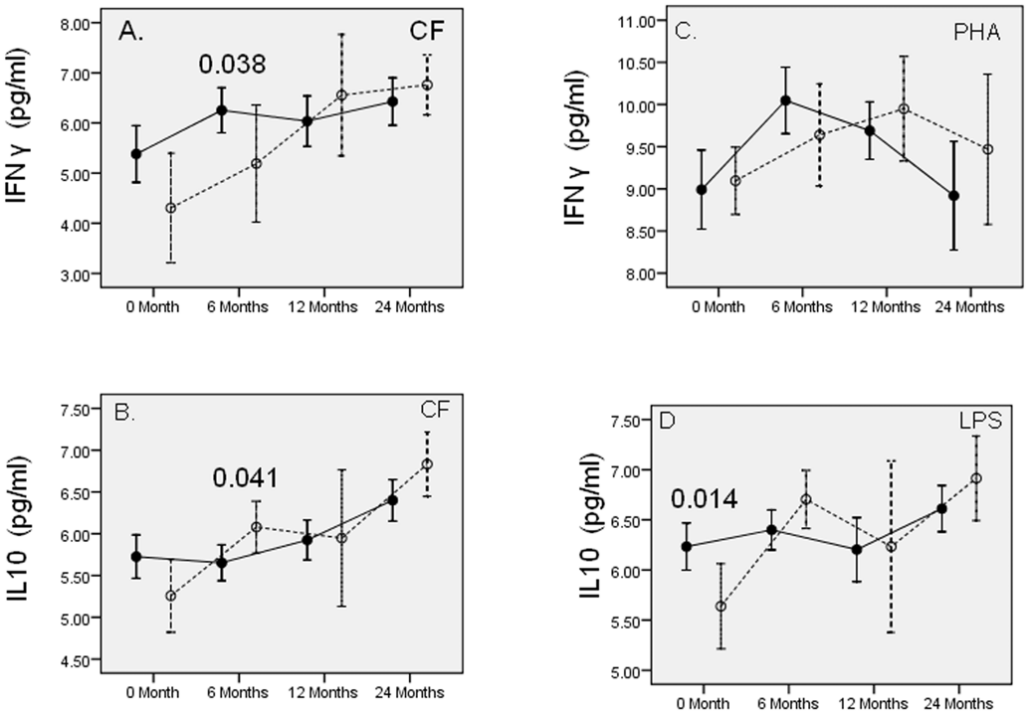
CF and mitogen induced IFN γ and IL-10 in TST+HC and TST-HC groups. Cytokine secretion was assessed in supernatants of stimulated whole blood cultures for all donors at 0, 6, 12 and 24 months. Previously infected (TST+HC = 54) HC are depicted as closed circles and solid lines; recently infected (TST-HC = 17) as open circles and broken lines. Concentrations of CF and mitogens, and days of stimulation are same as in Figure 2. Panel A and B show CF induced and panel C and D show mitogen induced cytokines. Months at which cytokines were assessed post exposure are shown on the X axis. The cytokine values are log _n_ transformed and means are shown on the Y axis. ANOVA test for repeated measures using GLM was carried to assess the differences in the two groups. Vertical line indicates the error bars (95% confidence intervals) at each time point. P values are shown for each time point. A p value<0.05 was considered significant.

**Figure 4:**
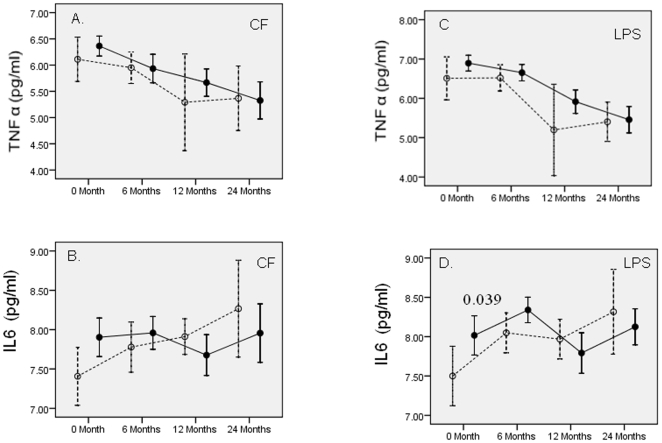
CF and mitogen induced TNF α and IL-6 in TST+HC and TST-HC groups. Previously infected (TST+HC = 54) HC are shown as closed circles and solid lines and recently infected (TST-HC = 17) by open circles and broken lines. Concentrations of CF and mitogens, and days of stimulation are same as in Figure 2. All other parameters are same as in Figure 3.

To identify the cytokine dynamics post exposure in the HC group with recent infection (TST-HC = 17) compared to the HC group with previous infection (TST+HC = 54) analysis of variance (ANOVA) was carried on repeated measures over a two year period. The reference group (TST+HC) had similar MTB exposure and demographics at recruitment including the presence of BCG scar (Table S1). Only donors sampled at all time points (54/70) were included in the analysis. Figure 3 (IFN γ and IL-10) and Figure 4 (TNF α and IL-6) show comparative profiles for mycobacterial antigen (CF) or mitogen (PHA or LPS) induced cytokine secretion.

Both groups showed dynamic changes in cytokines after acute exposure. Not surprisingly, CF induced IFN γ (Figure 3, panel A) showed significantly lower responses in recently infected compared to previously infected HC group at 6 months [TST-HC; lower IFN γ (p = 0.038)]. In response to PHA (Figure 3, panel C), the magnitude of IFN γ response did not differ between the two HC groups, but the timing of peak responses were different. The previously infected HC group showed peak IFN γ response at 6 months, while the recently infected HC group peaked at 12 months (Figure 3, panel C) suggesting that TST conversion may be related to the strength of mycobacterial specific T cell response rather than the overall strength of T cell activation. The trend with CF induced IL-10 (Figure 3, panel B) in the two groups was opposite to that observed with CF induced IFN γ with IL-10 responses being significantly higher in recently infected HC at 6 months (p = 0.041). Parallel trends were observed with LPS induced IL-10 (Figure 3, panel D). Both LPS and CF induced IL-10 showed a much more dynamic profile with greater variability in recently infected HC group (Table S2) compared to a relatively flat response over time in the previously infected HC group indicating that immune responses in newly infected HC take at least a year to stabilize.

No significant differences were noted with TNFα (Figure 4, panel A and C) or IL-6 (Figure 4, panel B and D) in response to CF or mitogens between recently infected compared to previously infected HC (Figure 4) The only exception was LPS induced IL-6 (Figure 4D) which was significantly higher (p = 0.039) in the previously infected HC group at 0 months compared to the HC group with recent infection at recruitment.

## Discussion

This is the first report on serial assessment of multiple biomarkers to identify changes associated with TST conversion in a recently MTB exposed cohort in a low HIV setting. Natural evolution of biomarkers post exposure was possible in those with recent and past infection as no prophylaxis was given for latent tuberculosis. The cohort remained disease free over a 4 year period suggesting that these biomarker profiles may represent protective profiles in the HC group post exposure. The strength of the study was therefore, careful selection of a cohort with no history of TB or of anti-tuberculous treatment in the past.

Variability in the duration and intensity of exposure can change the dynamics of immune parameters particularly with respect to innate responses that are activated early during infection. Both these parameters are very difficult to control in a high transmission setting. We have tried to limit this variability by including families where the symptoms in the index cases initiated no more than 3 months and the pulmonary disease was restricted to moderate lung disease. We have used MTB culture filtrate antigens which are comparable in activity to PPD antigens used in TST. In household contacts IFN γ secretion in response to culture filtrate antigens in stimulated whole blood have been recommended for early diagnosis of latent infection especially in a resource strapped countries [25]. When cytokines responses were compared in the exposed and the non exposed groups, elevated responses were observed with mycobacterial antigen induced TNFα and IL-10 which were most likely derived from innate cells. This was not surprising as mycobacterial proteins are known to be potent stimulators of macrophages [24], [33], [35], [and 36]. However, suppressed mycobacterial antigen induced IFN γ responses in the exposed HC group compared to the EC group was surprising. Pakistan has wide BCG vaccination coverage at birth (>70%) through the expanded program for immunization (EPI) [7] and a high prevalence of TST+ in the non exposed community control group may be due to either previous BCG vaccination [8] or booster effects with environmental mycobacteria [9]. As to why we did not observe such a booster effect in the recently exposed cohort may lie with CF induced IL-10 which was selectively raised in the exposed HC groups but not in the non exposed EC group. IL-10 is a down-regulatory cytokine and inhibits pro-inflammatory cytokines such as IFN γ [18], [19] and may be the reason for the observed lower CF induced IFN γ responses in the exposed HC group. The HC groups had been exposed to MTB for at least 3 months, which may be sufficient to activate the innate arm of the immune responses. CF induced IL-6 was the only cytokine which did not show differences between the HC and the EC groups. The HC groups stratified on the basis of TST status did not show significant differences (Mann Whitney U tests) in CF induced cytokine responses (Figure 3 &4). The differences between the non exposed EC and the exposed HC groups are therefore most likely due to recent MTB exposure.

In relation to TST conversion, the HC group did show an increase in CF induced IFN γ secretion post TST conversion. Similar increases in IFN γ to mycobacterial antigens have been reported post exposure [37] and post TST conversion to ESAT-6 which is a MTB specific antigen [27]. Antigen induced IFN γ has been reported to correlate with TST diameter [38], but the coordinate expression of CF induced IL-10 was surprising. A similar LPS induced IL-10 secretion was noted, suggesting an overall activation of macrophages post TST conversion. IL-10 may be elevated to reduce collateral tissue damage [19] in response to a heightened proinflammatory response during the early phase of infection. The time frame of TST conversion (6 months) and establishment of stable cytokine responses is also the time frame where the highest reported incidence of disease post MTB exposure was observed in this cohort [5]. Disease progression in this cohort (N = 7) was associated with depressed IFN γ/IL-10 ratio [5]. Surprisingly there was no concurrent elevation of TNF α post TST conversion. TNF α is prototypic marker of classically activated macrophages (CAM ϕ) while alternatively activated macrophages (AAM ϕ) secrete high levels of IL-10. [39]. Mycobacterial antigens can signal macrophages via the Toll like receptors [40] and recently TLR9 has been implicated in the activation of alternatively activated macrophages (AAM ϕ) [41]. It is therefore tempting to speculate that post TST conversion there is concurrent elevation of Th1 and AAM ϕ which down regulate CAM ϕ. Increase in IL-6 post TST conversion was also intriguing. Although the role of IL-6 in human tuberculosis is not clear, IL-6 has been shown to be elevated in established disease [33]. In the experimental IL-6 gene knockout mouse, a delay in early induction of IFN γ [23] as well a delay in the initial differentiation of Th1 cells, but not in expansion, has been reported [42].

To further understand the evolution of cytokines post MTB exposure, we compared the HC group with recent infection (TST converters) or past infection (TST+HC at recruitment). Again significant differences post exposure in the two groups, were noted only at 6 months. These results suggest that TST conversion may precede latency and stable granuloma formation as cytokine responses in the two groups (recent infection Vs previous infection) become comparable beyond 12 months. The cytokines likely to play a role in TST conversion such as IFN γ show a plateau beyond 12 months while IL-10 continues to increase in both groups beyond 12 months and may play a greater role in establishing dormancy and latency. These findings therefore supports the notion that latent tuberculosis actually represents a subclinical spectrum which can only be defined by the host immune response [43] and is supported by the variability of responses in recently infected groups while previously infected groups represent a more stable host response.

Our studies therefore suggest that while IFN γ may play an important role in resolution of infection and TST conversion, IL-10 may be critical in dampening the pro inflammatory arm of the immune response as well as in initiation of dormancy during the later phase of infection. The current study therefore, raises important questions with regards to the source of various biomarkers and their role in establishing TST conversion, dormancy and latency.

## Supporting Information

Table S1 Intensity and duration of exposure to M.tuberculosis in Household Contacts at Recruitment. a Cough and or low grade fever and or weight loss. b Intensity of Acid Fast Bacilli in sputum smear. c Determined by radiology (According to Crofton et al 1990[30] d ATT (days) started prior recruitment. TST+HC were not given prophylactic ATT. e # HC in the family. # TST-HC in the HC within each family is shown in brackets. * repeat TST available at 24 months only. Cytokine levels were available at all time points (0, 6, 12 and 24 months) on 77 HC (TST+ = 54; TST- = 23). Secondary cases (N = 8) diagnosed over 4 years follow up, and contacts previously treated (N = 5) were excluded. § One co-prevalent case (on ATT) in the family BCG scar was present in 40% (38/94) HC. Among BCG scar positive HC, 71% were TST positive, and in BCG scar negative HC 78.5% were TST positive. Reference: Crofton J (1990) Clinical features of tuberculosis. In: Seton D, Gordon A, editors. Crofton and Douglas Respiratory Diseases. London: Blackwell Scientific. pp. 395–421.
(0.05 MB DOC)

Table S2 Repeated measure on TST+ HC and TST- HC. Intergroup comparison of cytokine profiles in the HC group with previous infection (TST+HC at intake) with recent infection (TST-HC at intake) in response to mycobacterial antigens (CF) and mitogens (PHA or LPS). This is a companion table for Figures 3 and 4. ANOVA test for repeated measures using GLM was carried to assess the differences in the two groups. The cytokine values are log n transformed and the estimated marginal means, SEM, β estimates, p value and 95% CI for each of the stimulant and cytokines shown in Figure 3 and Figure 4. A p value<0.05 was considered significant.
(0.12 MB DOC)
