# Supplementary material for: Biomarker Changes Associated with Tuberculin Skin Test (TST) Conversion: A Two-Year Longitudinal Follow-Up Study in Exposed Household Contacts
Source: PLoS One. 2009 Oct 14;4(10):e7444. doi: 10.1371/journal.pone.0007444 (PMC2758599; doi:10.1371/journal.pone.0007444)
Supplement: Table S1 — Intensity and duration of exposure to M.tuberculosis in Household Contacts at Recruitment. a Cough and or low grade fever and or weight loss. b Intensity of Acid Fast Bacilli in sputum smear. c Determined by radiology (According to Crofton et al 1990[30] d ATT (days) started prior recruitment. TST+HC were not given prophylactic ATT. e # HC in the family. # TST-HC in the HC within each family is shown in brackets. * repeat TST available at 24 months only. Cytokine levels were available at all time points (0, 6, 12 and 24 months) on 77 HC (TST+ = 54; TST- = 23). Secondary cases (N = 8) diagnosed over 4 years follow up, and contacts previously treated (N = 5) were excluded. § One co-prevalent case (on ATT) in the family BCG scar was present in 40% (38/94) HC. Among BCG scar positive HC, 71% were TST positive, and in BCG scar negative HC 78.5% were TST positive. Reference: Crofton J (1990) Clinical features of tuberculosis. In: Seton D, Gordon A, editors. Crofton and Douglas Respiratory Diseases. London: Blackwell Scientific. pp. 395–421. (0.05 MB DOC) [file pone.0007444.s001.doc]

**Table S1. Intensity and duration of exposure to *M.tuberculosis*** in Household Contacts at Recruitment

| Family# | Index case | ^a^ history of symptoms(days) | ^b^AFB (Sputum smear) | ^c^ extent of lung involvement | ^d^ dur. of ATT (days) | ^e^ Total HC (TST-HC) |
| --- | --- | --- | --- | --- | --- | --- |
| 1 | TB267 | 30 | ++ | PMN | 10 | 5 (3)* |
| 2 | TB268 | 60 | ++ | PMD | 18 | 5 (3)* |
| 3 | TB269 | 30 | ++ | PMD | 1 | 3 (0) |
| 4 | TB270 | 90 | ++ | PMD | 6 | 5 (0) |
| 5 | TB271 | 60 | ++ | PMD | 6 | 3 (0) |
| 6 | TB272 | 90 | ++ | PMD | 7 | 2 (0) |
| 7 | TB273 | 60 | ++ | PMD | 2 | 1 (0) |
| 8 | TB281 | 30 | ++ | PMD | 1 | 3 (0) |
| 9^§^ | TB282 | 90 | ++ | PMD | 6 | 4 (2) |
| 10 | TB283 | 90 | ++ | PMD | 9 | 6 (0) |
| 11 | TB285 | 60 | ++ | PMN | 4 | 6 (2) |
| 12^§^ | TB286 | 60 | ++ | PAD | 3 | 5 (0) |
| 13 | TB290 | 120 | ++ | PMD | 2 | 5 (0) |
| 14 | TB291 | 30 | ++ | PMD | 4 | 6 ((2) |
| 15 | TB292 | 60 | ++ | PMD | 2 | 5 (0) |
| 16 | TB298 | 90 | ++ | PAD | 13 | 8 (2) |
| 17 | TB299 | 90 | ++ | PMD | 8 | 6 (3) |
| 18 | TB300 | 90 | ++++ | PMD | 24 | 7 (4) |
| 19 | TB301 | 120 | ++ | PMN | 4 | 3 (0) |
| 20 | TB302 | 90 | ++ | PMD | 3 | 6 (2) |
