# Supplementary material for: Biomarker Changes Associated with Tuberculin Skin Test (TST) Conversion: A Two-Year Longitudinal Follow-Up Study in Exposed Household Contacts
Source: PLoS One. 2009 Oct 14;4(10):e7444. doi: 10.1371/journal.pone.0007444 (PMC2758599; doi:10.1371/journal.pone.0007444)
Supplement: Table S2 — Repeated measure on TST+ HC and TST- HC. Intergroup comparison of cytokine profiles in the HC group with previous infection (TST+HC at intake) with recent infection (TST-HC at intake) in response to mycobacterial antigens (CF) and mitogens (PHA or LPS). This is a companion table for Figures 3 and 4. ANOVA test for repeated measures using GLM was carried to assess the differences in the two groups. The cytokine values are log n transformed and the estimated marginal means, SEM, β estimates, p value and 95% CI for each of the stimulant and cytokines shown in Figure 3 and Figure 4. A p value<0.05 was considered significant. (0.12 MB DOC) [file pone.0007444.s002.doc]

**Table S2. Repeated measure on TST+ HC and TST- HC**

1. **IFNγ (PHA)**

| Parameter | Gp ID | Mean | SEM | Beta estimate | Std Error | P value | 95% CI | |
| --- | --- | --- | --- | --- | --- | --- | --- | --- |
|  |  |  |  |  |  |  | Lower | Upper |
| 0 month | TST-HC | 9.095 | 0.386 | 0.111 | 0.442 | 0.802 | -0.771 | 0.993 |
|  | TST+HC | 8.984 | 0.215 |  |  |  |  |  |
| 6month | TST-HC | 9.640 | 0.339 | -0.461 | 0.388 | 0.238 | -1.234 | 0.312 |
|  | TST+HC | 10.100 | 0.188 |  |  |  |  |  |
| 12month | TST-HC | 9.950 | 0.305 | 0.214 | 0.349 | 0.541 | -0.482 | 0.911 |
|  | TST+HC | 9.735 | 0.170 |  |  |  |  |  |
| 24 month | TST-HC | 9.469 | 0.545 | 0.567 | 0.623 | 0.366 | -0.675 | 1.810 |
|  | TST+HC | 8.902 | 0.303 |  |  |  |  |  |

1. **IFNγ (CF)**

| Parameter | Gp ID | Mean | SEM | Beta estimate | Std Error | P value | 95% CI | |
| --- | --- | --- | --- | --- | --- | --- | --- | --- |
|  |  |  |  |  |  |  | Lower | Upper |
| 0 month | TST-HC | 4.305 | 0.515 | -1.034 | 0.586 | .082 | -2.203 | 0.134 |
|  | TST+HC | 5.407 | 0.286 |  |  |  |  |  |
| 6month | TST-HC | 5.191 | 0.431 | -1.074 | 0.507 | **.038** | -2.085 | -0.063 |
|  | TST+HC | 6.350 | 0.239 |  |  |  |  |  |
| 12month | TST-HC | 6.556 | 0.479 | 0.474 | 0.554 | 0.395 | -0.630 | 1.578 |
|  | TST+HC | 6.115 | 0.266 |  |  |  |  |  |
| 24 month | TST-HC | 6.762 | 0.394 | 0.102 | 0.364 | 0.781 | -0.624 | 0.828 |
|  | TST+HC | 6.456 | 0.219 |  |  |  |  |  |

1. **IL10 (LPS)**

| Parameter | Gp ID | Mean | SEM | Beta estimate | Std Error | P value | 95% CI | |
| --- | --- | --- | --- | --- | --- | --- | --- | --- |
|  |  |  |  |  |  |  | Lower | Upper |
| 0 month | TST-HC | 5.639 | 0.210 | -0.606 | 0.241 | **.014** | -1.087 | -0.126 |
|  | TST+HC | 6.245 | 0.117 |  |  |  |  |  |
| 6month | TST-HC | 6.704 | 0.172 | 0.279 | 0.197 | 0.162 | -0.114 | 0.671 |
|  | TST+HC | 6.425 | 0.096 |  |  |  |  |  |
| 12month | TST-HC | 6.292 | 0.285 | 0.075 | 0.326 | 0.819 | -0.576 | 0.725 |
|  | TST+HC | 6.217 | 0.158 |  |  |  |  |  |
| 24 month | TST-HC | 6.915 | 0.208 | 0.344 | 0.238 | 0.154 | -0.131 | 0.819 |
|  | TST+HC | 6.571 | 0.116 |  |  |  |  |  |

(iv) **IL10 (CF)**

| Parameter | Gp ID | Mean | SEM | Beta estimate | Std Error | P value | 95% CI | |
| --- | --- | --- | --- | --- | --- | --- | --- | --- |
|  |  |  |  |  |  |  | Lower | Upper |
| 0 month | TST-HC | 5.258 | 0.225 | -0.435 | 0.257 | 0.095 | -0.948 | 0.077 |
|  | TST+HC | 5.693 | 0.125 |  |  |  |  |  |
| 6month | TST-HC | 6.080 | 0.182 | 0.433 | 0.208 | **.041** | 0.018 | 0.849 |
|  | TST+HC | 5.646 | 0.101 |  |  |  |  |  |
| 12month | TST-HC | 5.947 | 0.265 | 0.041 | 0.303 | .892 | -0.562 | 0.645 |
|  | TST+HC | 5.906 | 0.147 |  |  |  |  |  |
| 24 month | TST-HC | 6.834 | 0.219 | 0.451 | 0.251 | .076 | -0.049 | 0.951 |
|  | TST+HC | 6.383 | 0.122 |  |  |  |  |  |

(v) **TNFα (LPS**)

| Parameter | GpID | Mean | SEM | Beta estimate | Std Error | p value | 95% CI | |
| --- | --- | --- | --- | --- | --- | --- | --- | --- |
|  |  |  |  |  |  |  | Lower | Upper |
| 0 month | TST-HC | 6.509 | 0.200 | -0.385 | 0.229 | 0.097 | -0.841 | 0.071 |
|  | TST+HC | 6.894 | 0.111 |  |  |  |  |  |
| 6month | TST-HC | 6.520 | 0.181 | -0.133 | 0.207 | 0.523 | -0.545 | 0.279 |
|  | TST+HC | 6.653 | 0.100 |  |  |  |  |  |
| 12month | TST-HC | 5.197 | 0.352 | -0.718 | 0.402 | 0.078 | -1.521 | 0.084 |
|  | TST+HC | 5.916 | 0.195 |  |  |  |  |  |
| 24 month | TST-HC | 5.404 | 0.288 | -0.053 | 0.329 | 0.872 | -0.710 | 0.604 |
|  | TST+HC | 5.457 | 0.160 |  |  |  |  |  |

(vi) **TNFα (CF**)

| Parameter | GpID | Mean | SEM | Beta estimate | Std Error | p value | 95% CI | |
| --- | --- | --- | --- | --- | --- | --- | --- | --- |
|  |  |  |  |  |  |  | Lower | Upper |
| 0 month | TST-HC | 6.110 | 0.178 | -0.259 | 0.204 | 0.209 | -0.666 | 0.149 |
|  | TST+HC | 6.369 | 0.099 |  |  |  |  |  |
| 6month | TST-HC | 5.950 | 0.223 | 0.032 | 0.256 | 0.900 | -0.478 | 0.542 |
|  | TST+HC | 5.918 | 0.124 |  |  |  |  |  |
| 12month | TST-HC | 5.291 | 0.292 | -0.384 | 0.335 | 0.254 | -1.052 | 0.283 |
|  | TST+HC | 5.676 | 0.163 |  |  |  |  |  |
| 24 month | TST-HC | 5.368 | 0.311 | 0.072 | 0.356 | 0.841 | -0.639 | 0.782 |
|  | TST+HC | 5.296 | 0.173 |  |  |  |  |  |

(vii) **IL6 (LPS**)

| Parameter | GpID | Mean | SEM | Beta estimate | Std Error | p value | 95% CI | |
| --- | --- | --- | --- | --- | --- | --- | --- | --- |
|  |  |  |  |  |  |  | Lower | Upper |
| 0 month | TST-HC | 7.500 | 0.214 | -0.516 | 0.245 | **0.039** | -1.004 | -0.028 |
|  | TST+HC | 8.016 | 0.119 |  |  |  |  |  |
| 6month | TST-HC | 8.049 | 0.140 | -0.290 | 0.160 | 0.074 | -0.610 | 0.029 |
|  | TST+HC | 8.339 | 0.078 |  |  |  |  |  |
| 12month | TST-HC | 7.969 | 0.210 | 0.176 | 0.241 | 0.466 | -0.304 | 0.657 |
|  | TST+HC | 7.792 | 0.117 |  |  |  |  |  |
| 24 month | TST-HC | 8.316 | 0.217 | 0.191 | 0.249 | 0.446 | -0.305 | 0.687 |
|  | TST+HC | 8.126 | 0.121 |  |  |  |  |  |

(viii) **IL6(CF**)

| Parameter | GpID | Mean | SEM | Beta estimate | Std Error | p value | 95% CI | |
| --- | --- | --- | --- | --- | --- | --- | --- | --- |
|  |  |  |  |  |  |  | Lower | Upper |
| 0 month | TST-HC | 7.407 | 0.208 | -0.465 | 0.238 | 0.054 | -0.940 | 0.009 |
|  | TST+HC | 7.873 | 0.116 |  |  |  |  |  |
| 6month | TST-HC | 7.778 | 0.174 | -0.152 | 0.199 | 0.448 | -0.549 | 0.245 |
|  | TST+HC | 7.930 | 0.097 |  |  |  |  |  |
| 12month | TST-HC | 7.912 | 0.210 | 0.245 | 0.240 | 0.311 | -0.234 | 0.723 |
|  | TST+HC | 7.667 | 0.117 |  |  |  |  |  |
| 24 month | TST-HC | 8.267 | 0.324 | 0.309 | 0.371 | 0.407 | -0.430 | 1.048 |
|  | TST+HC | 7.958 | 0.180 |  |  |  |  |  |
